# Regulation of Myelination by Exosome Associated Retinoic Acid Release from NG2-Positive Cells

**DOI:** 10.1523/JNEUROSCI.2922-18.2019

**Published:** 2019-04-17

**Authors:** Maria B. Goncalves, Yue Wu, Earl Clarke, John Grist, Carl Hobbs, Diogo Trigo, Julian Jack, Jonathan P.T. Corcoran

**Affiliations:** The Wolfson Centre for Age-Related Diseases, King's College London, Guy's Campus, London SE1 1UL, United Kingdom

**Keywords:** decorin, exosome, myelination, NG2^+^ cells, RARβ, RARα, retinoic acid

## Abstract

In the CNS, oligodendrocytes are responsible for myelin formation and maintenance. Following spinal cord injury, oligodendrocyte loss and an inhibitory milieu compromise remyelination and recovery. Here, we explored the role of retinoic acid receptor-beta (RARβ) signaling in remyelination. Using a male Sprague Dawley rat model of PNS-CNS injury, we show that oral treatment with a novel drug like RARβ agonist, C286, induces neuronal expression of the proteoglycan decorin and promotes myelination and differentiation of oligodendrocyte precursor cells (NG2^+^ cells) in a decorin-mediated neuron–glia cross talk. Decorin promoted the activation of RARα in NG2^+^ cells by increasing the availability of the endogenous ligand RA. NG2^+^ cells synthesize RA, which is released in association with exosomes. We found that decorin prevents this secretion through regulation of the EGFR–calcium pathway. Using functional and pharmacological studies, we further show that RARα signaling is both required and sufficient for oligodendrocyte differentiation. These findings illustrate that RARβ and RARα are important regulators of oligodendrocyte differentiation, providing new targets for myelination.

**SIGNIFICANCE STATEMENT** This study identifies novel therapeutic targets for remyelination after PNS-CNS injury. Pharmacological and knock-down experiments show that the retinoic acid (RA) signaling promotes differentiation of oligodendrocyte precursor cells (OPCs) and remyelination in a cross talk between neuronal RA receptor-beta (RARβ) and RARα in NG2^+^ cells. We show that stimulation of RARα is required for the differentiation of OPCs and we describe for the first time how oral treatment with a RARβ agonist (C286, currently being tested in a Phase 1 trial, ISRCTN12424734) leads to the endogenous synthesis of RA through retinaldehyde dehydrogenase 2 (Raldh2) in NG2 cells and controls exosome-associated-RA intracellular levels through a decorin–Ca^2+^ pathway. Although RARβ has been implicated in distinct aspects of CNS regeneration, this study identifies a novel function for both RARβ and RARα in remyelination.

## Introduction

After spinal cord injury (SCI), there is widespread oligodendrocyte cell death and demyelination. Remyelination is important to restore reliable axonal transmission (Smith et al., 1979) and protect axons from degeneration (Irvine and Blakemore, 2008). When demyelinating lesions occur, newly generated oligodendrocytes (OLs), mostly derived from oligodendrocyte precursor cells (OPCs), can repair or replace damaged myelin (Salgado-Ceballos et al., 1998; Zawadzka et al., 2010). Nevertheless, due to external and intrinsic inhibitory cues, the amount of new myelin is unable to cover all exposed axons, generating a negative myelin balance that increases the number of naked axons and thereby results in degeneration and sensory and motor deficits in residual nerves. The microenvironment after SCI is not particularly favorable to remyelination: chondroitin sulfate proteoglycans (CSPGs) in the extracellular matrix can inhibit remyelination by blocking OPC migration (Siebert and Osterhout, 2011), the glial scar hinders OPC proliferation and migration, and downregulation of nutrients and growth factors also limit remyelination (Meletis et al., 2008; Gauthier et al., 2013; Lukovic et al., 2015). Here, we sought to identify mechanisms and their putative therapeutic targets that can relieve inhibition to myelination and promote proliferation and differentiation of OPCs.

Several studies have shown that retinoic acid receptor-beta (RARβ) activation is beneficial in a variety of CNS injuries (Dmetrichuk et al., 2005; Wong et al., 2006; Yip et al., 2006; Agudo et al., 2010; Puttagunta et al., 2011; Koriyama et al., 2013; Hernández-Pedro et al., 2014; Goncalves et al., 2015, 2018). We have previously shown that stimulation of the RARβ signaling pathway in an animal model of dorsal root avulsion (DRA)-induced recovery of motor and sensory functions and this was accompanied by an increase in remyelination across the dorsal root entry zone (DREZ) (Goncalves et al., 2015).

Here, we show that the RA signaling pathway promotes remyelination in a twofold way: eliminating the inhibitory CSPGs from the extracellular milieu and driving the differentiation of NG2^+^ cells into myelinating OLs. We found that activation of neuronal RARβ results in the upregulation of decorin. This small leucine proteoglycan that can suppress the core protein levels of the CSPGs at sites of acute rat SCI resulting from suppression of CSPG synthesis and/or a potential increase in CSPG degradation (Davies et al., 2004, 2006). Furthermore, decorin has also been shown to inhibit the activity of transforming growth factor β (TGFβ) (Yamaguchi et al., 1990) and the epidermal growth factor receptor (EGFR) (Csordás et al., 2000), both of which regulate the synthesis of axon growth inhibitory CSPGs (Asher et al., 2000; Dobbertin et al., 2003). Additionally, we show that activation of the RARα in NG2^+^ cells, which is expressed in OPCs (Tripathi and McTigue, 2007; Barnabe-Heider et al., 2010; Hughes et al., 2013), drives their differentiation into myelin-producing oligodendrocytes *in vivo. In vitro*, deletion of RARα was sufficient to abrogate oligodendrocyte genesis from NG2^+^ cells. We further show that endogenous RA is essential to drive RARα signaling *in vivo* because the transduction of a lentivirus overexpressing Raldh2 silencing RNA (shRNA) in RARβ-agonist-treated, avulsed rats prevented myelination. Likewise, treatment with disulfiram, an aldehyde dehydrogenase 2 inhibitor (Lipsky et al., 2001), yielded similar results. RARβ signaling results in the increased expression of Raldh2 in NG2^+^ cells responsible for the synthesis of RA, which is secreted in association with exosomes during axonal regeneration (Goncalves et al., 2018). Here, we found that decorin reduces intracellular Ca^2+^ in NG2^+^ cells cultured in the presence of retinal and this prevents the release of exosome-associated RA. The same effect was seen with an intracellular Ca^2+^ chelator.

Collectively our results demonstrate the dual decorin-mediated effect of RA signaling in remyelination after SCI and sheds novel insights into the cross talk between RARβ and RARα endogenous pathways. Additionally, we herein identify two therapeutic targets, RARα and RARβ, of interest for the treatment of demyelination.

## Materials and Methods

### 

#### 

##### Surgery and drug treatments.

All surgeries, behavioral testing, and analysis were performed using a randomized block design and in a blinded fashion. Allocation concealment was performed by having the treatments stocks coded by a person independent of the study. The blinded treatment code for each rat was drawn at random from a hat without replacement. Codes were only broken after the end of the study. All procedures were in accordance with the UK Home Office guidelines and Animals (Scientific Procedures) Act of 1986. All animal care and experimental procedures complied with the Animals (Scientific Procedures) Act of 1986 of the UK Parliament, Directive 2010/63/EU of the European Parliament, and the *Guide for the Care and Use of Laboratory Animals* published by the National Institutes of Health (NIH Publication No. 85-23, revised 1996). Animal studies are reported in compliance with the ARRIVE guidelines (Kilkenny et al., 2010; McGrath and Lilley, 2015).

Animals were housed in groups of three to four in Plexiglas cages with tunnels and bedding, on a 12/12 h light/dark cycle and had access to food and water *ad libitum*.

For the DRA model, male Sprague Dawley rats weighing 220–250 g (*n* = 8 per treatment group for each set of experiments) were anesthetized with an intramuscular injection of 60 mg/kg ketamine and 0.4 mg/kg metidomidine and their backs were shaved and aseptically prepared. After opening the skin and muscle layers, C5–C8 and T1 dorsal roots were cut flush with the spinal cord (SC) surface. The cut ends of the dorsal roots were subsequently introduced through slits in the pia mater and positioned superficially in the SC adjacent to where they had been cut (Goncalves et al., 2015). During the surgery, the rats were placed on a controlled heating pad to maintain temperature at 37 ± 1°C. After the surgery, the rats were hydrated with physiological saline (2 ml, s.c.). Anesthesia was reversed with an intramuscular injection of 0.05 ml (1 mg/kg) atipamezole hydrochloride (Antisedan; Pfizer Animal Health). Animals were kept in a heated recovery box until fully conscious and analgesia (buprenorphine, 0.01 mg/kg, subcutaneously) was given after suturing and recovery. All rats survived this surgery.

For Raldh2 loss-of-function studies, 5 μl of lentivirus (titer: 3.67 × 10^8^ TU/ml) was injected manually at the DREZ of the severed sensory roots using a 20 μl Hamilton syringe at 0.5 μl min^−1^ and the needle was left in place for 5 min to limit diffusion through the needle tract. Lentiviruses were provided by GeneCopoeia. The shRNA to rat Raldh-2 sequence gatccgggcatagacaagattgcattctcaagaggaatgcaatcttgtctatgccttttttg was ligated into psi-LVRH1GH. The lentiviral (LV) particles were generated by following a standardized protocol using highly purified plasmids and EndoFectin-Lenti and TiterBoost reagents. The LV transfer vector was cotransfected into 293Ta cells with the Lenti-Pac HIV packaging mixture.

##### Drug treatments for *in vivo* studies.

Rats were treated with: vehicle or the novel selective RARβ agonist C286 (synthesized by Sygnature Chemical Services) given by oral gavage 3 times a week for 4 weeks at 3 mg/kg (*n* = 8 per treatment group) or with the aldehyde dehydrogenase inhibitor disulfiram (10 mg/kg) (Mittal et al., 2014) by intraperitoneal injection 3 times a week for 4 weeks. C286 has a high potency at RARβ (similar potency to all-*trans*-RA) and behaves as a full agonist, showing a half-maximal effective concentration (EC_50_) of 1.94 nm at the mouse RARβ receptor, a selectivity for RARβ over RARα of 13.4, and a selectivity for RARβ over RARγ of 5.6-fold.

Animals were culled after 4 weeks of treatment. Rats were perfused transcardially with heparinized 0.9% NaCl solution and 4% PFA. The cervical cords with attached DRGs were dissected, rapidly removed, and postfixed with 4% PFA for at least 2 d at room temperature. Tissue was then embedded in paraffin wax and 5 μm longitudinal sections cut throughout each block. Sets of consecutive sections comprising the lesioned area and taken from equivalent regions of the spinal cord were used for immunostaining.

##### Histological and quantitative analysis of proteins in the rat tissue.

Quantification of immunofluorescence CS56, decorin, olig1, myelin-associated protein (MAG), NG2, Raldh2, proteolipid protein (PLP), RARα, and RARβ was done as described previously (Herrmann et al., 2010).

In brief, positively stained areas were quantified as the pixels of immunoreactivity above a threshold level per unit area. The threshold value was set to include fluorescent positive signal and to exclude background staining. Threshold values for a given section and stain remained the same throughout the study. The number of pixels was measured in a 300 μm^2^ area comprising the DREZ of the implanted severed dorsal roots and the contiguous spinal cord. At least 10 sections per rat were used for these quantifications.

##### Primary neuronal cell cultures.

Mouse primary cortical neurons were prepared as described previously (Goncalves et al., 2013). Cells were plated onto 5 μg/ml poly-d-lysine-coated 100 mm dishes at a density of 6 × 10^6^ cells/dish. Cells were cultured in Neurobasal medium (Invitrogen) containing 2% (v/v) B27 serum-free supplement, 2 mm l-glutamine, 1.5% glucose, penicillin (100 units/ml), and streptomycin (100 μg/ml) at 37°C in a humidified atmosphere of 5% CO_2_/95% air. Cultures were used after 1 week and were >98% neurons judged by βIII-tubulin staining. Treatments were as follows: mouse primary cortical cultures (0 d *in vitro*) were treated with vehicle or 10^−7^ m C286 in the presence or absence of decorin neutralizing antibody (NAb,5 μg/ml) for 72 h. The conditioned medium was then collected and added to astrocyte cultures for another 72 h as indicated. Culture conditions were three wells per treatment performed three times independently.

##### Astrocyte cultures.

Primary mixed glial cultures were prepared as described previously (Goncalves et al., 2013) using a modified protocol. Briefly, mixed glial cultures were obtained from the cortices of postnatal day 5 (P5) to P8 mice. Cultures were maintained at 37°C (5% CO_2_/95% O_2_) in DMEM/F12 medium containing 15% fetal bovine serum (FBS) (Invitrogen) and 1% penicillin–streptomycin (Sigma-Aldrich) for 10–14 d. Microglial and NG2^+^ cells were then harvested by forceful shaking for 1 min by hand. After harvesting of microglia and NG2^+^ cells, done every 3 d for 9 d, 0.25% trypsin EDTA was added to each flask for 5 min. Cells were then centrifuged at 1000 rpm for 5 min and plated on poly-d-lysine-coated 8-well chamber slides in 5% FBS. To obtain reactive astrocytes, cells were treated with 100 ng/ml lipopolysaccharides (LPS) for 24 h (Tarassishin et al., 2014) before any other treatment was initiated. Treatments were as follows: vehicle, C286 (10^−7^ m), decorin (20 μg/ml, Sigma D8428), neuronal conditioned media from neurons (that had been treated as indicated), and in the presence or absence of decorin NAb (5 μg/ml). Astrocytes were treated for 72 h, after which cells were fixed and immunoassayed.

##### NG2^+^ cell culture.

Primary mixed glial cultures were prepared as described previously (Goncalves et al., 2013) using a modified protocol. Briefly, mixed glial cultures were obtained from the cortices of P5 mice. Cultures were maintained at 37°C (5% CO_2_/95% O_2_) in DMEM/F12 medium supplemented with 15% fetal bovine serum (FBS) (Invitrogen, Life Technologies), 9 mm glucose (Sigma-Aldrich), 2 mm l-glutamine (Invitrogen), and 1% penicillin–streptomycin (Invitrogen) on poly-d-lysine (PDL, 5 μg/ml, Sigma-Aldrich) precoated 75 cm^2^ flasks for 10 d. Microglial cells were then harvested by forcefully tapping at the side of the flasks six times. Medium was collected and centrifuged at 1000 rpm for 5 min. The cells were plated in a 75 cm^2^ flask at 37°C for 30 min to allow microglial cells to attach. Medium was collected again and centrifuged at 1000 rpm for 5 min. Cells were resuspended in NG2 growth medium [DMEM/F12 supplemented with 1% N2, 2% B27, 2 mm l-glutamine, 1% penicillin–streptomycin, 10 ng/ml FGF2 (R&D Systems) and 10 ng/ml PDGF-AA (Cell Guidance Systems)] and plated in PDL-precoated culture vessels at the density of 30,000 cells/cm^2^ for NG2–neuron coculture or 70,000 cells/cm^2^ for siRNA transfection. The purity of the NG2 cultures was confirmed by routine immunostaining, with ∼95% of the cells being NG2^+^. For the F9-RARE LacZ reporter assay, 99% confluent NG2^+^ cells were cultured in PLD-precoated 75 cm^2^ Thermo Scientific/Nunc cell culture flasks with filter caps for 3 d as described.

Cell cultures were treated as described with: dimethylsulfoxide (DMSO) was used as a vehicle and the retinoids were used in 1000× stock concentrations in DMSO. The retinoids used have been described previously (Goncalves et al., 2009). The retinoids (final concentration 10^−7^ m) used were retinal (Sigma-Aldrich), AM580 an RARα-selective agonist (Goncalves et al., 2009) and C286 an RARβ-selective agonist (Goncalves et al., 2019), which were synthesized by Sygnature Chemical Services. A RARα-selective agonist (AM 580) and a RARβ-selective agonist (C286), retinal and were all synthesized by Sygnature Chemical Services and used at 10^−7^ m. Other culture treatments were as follows: the permeable calcium chelator acetoxymethyl-1,2-bis (2-aminophenoxy) ethic acid (BAPTA/AM, Tocris Bioscience, 2.5 μm) (Wie et al., 2001), the exosome inhibitor GW4869 at 10^−6^ m, and decorin (20 μg/ml, Sigma-Aldrich, D8428). For Ca^2+^ assays, Fluo-4AM was used according to the manufacturer's instructions. In brief, Fluo-4AM was mixed with the non-ionic detergent Pluronic F-127 (Invitrogen) to give a stock solution with the concentration of 0.5 mm Fluo-4 and 10% Pluronic F-127, which was then added for 1 h to the cultures at a final concentration of 1 μm. Culture conditions were three wells per treatment performed three times.

##### Transfection of NG2^+^ cells.

Mouse NG2^+^ cells were transfected with mouse RARα siRNA with the duplex sequence rGrCrArArGrUrArCrArCrUrArCrGrArArCrArArCrArGrCTC or scrambled mouse siRNA (OriGene) using Lipofectamine 2000 transfection reagent (Invitrogen) according to the manufacturer's instructions. Briefly, mouse NG2^+^ cells were plated in PDL-coated culture vessels with density of 70,000 cells/cm^2^ in NG2 growth medium. The next day, culture medium was changed to NG2 growth medium without penicillin–streptomycin 2 h before transfection; 0.5 μl of Lipofectamine 2000 transfection reagent was used for every 6.25 pmol of siRNA. Six hours after transfection, cells were applied to different treatment conditions.

##### qRT-PCR.

Total RNA was extracted from siRNA-transfected NG2^+^ cells treated with vehicle using the RNeasy mini kit (Qiagen) 24, 48, and 72 h after transfection. Five hundred nanogram total RNA was used for first-strand cDNA synthesis with high-capacity cDNA reverse transcription kit (Applied Biosystems). qRT-PCR was performed using LightCycler 480 SYBR Green I master mixture (Roche) and gene-specific primers on a LightCycler 480 II (Roche). Primers used were as follows: for GAPDH, forward primer 5′-CGTAGACAAAATGGTGAAGGT-3′, reverse primer 5′-GACTCCACGACACTCAGC-3′; for RARα, forward primer 5′-AGATAGTACCCAGCCCTCC-3′, reverse primer 5′-TTCTTCTGGATGCTTCGTCG-3′. Levels of RARα mRNA were normalized to the reference gene GAPDH and relative change in mRNA levels following treatment was calculated using the 2^−ΔΔCt^ method.

##### Quantification of immunofluorescence in astrocyte cultures and decorin in neuronal cultures.

Ten random fields per treatment condition were captured using a 63× oil-immersion Aprochromat objective (Carl Zeiss) and the total pixel values for each antibody immunofluorescence staining (CD44, CSPG, and decorin) were measured using Axiovision software. The experimental conditions were done in triplicate and repeated three times independently and the quantifications were done by an operator blinded to the treatments.

##### Quantification of immunofluorescence in NG2^+^ cell cultures.

For quantification of oligodendrocyte-specific protein (OSP), NG2, EGFR, intracellular Ca^2+^ dye (Fluo-4AM), and RARα in NG2^+^ cell cultures, high-magnification images were obtained and mean intensity values of immunoreactivity were normalized for DAPI staining in each field. Five fields per culture condition, which were done in triplicate, were taken by a blinded operator and this was repeated independently three times.

##### F9-RARE LacZ reporter assay.

Murine F9 embryonal carcinoma cells, which stably express an RARβ2-promoter construct, were used as described previously (Sonneveld et al., 1999). Cells were scored on a blue versus not blue basis and the number or percentage of F9-RARE LacZ-positive cells were determined from five random fields from three independent experiments (Sonneveld et al., 1999).

##### Exosomes.

Exosomes were prepared from conditioned media from cell cultures using total exosome isolation (TEI) reagent (Invitrogen, Life Technologies) in accordance with the manufacturer's instructions. Conditioned media were centrifuged at 2000 × g for 30 min at 4°C to remove cells and cell debris and the resulting supernatants were mixed with 0.5 volumes of TEI reagent and centrifuged at 10,000 × *g* for 1 h at 4°C following overnight 4°C incubation. Exosome pellets were twice washed by resuspension in ice-cold PBS followed by centrifugation at 100,000 × *g* for 1 h at 4°C. Unused intact exosomes were stored at −80°C as PBS suspensions. For F9-RARE-lacZ RA reporter cell experiments, a pool of exosomes isolated from a 75 cm^2^ Thermo Scientific/Nunc cell culture treated flasks with filter caps of 99% confluent NG2^+^ cells were used.

##### Western blotting.

Proteins were separated by SDS-PAGE on 10% (w/v) polyacrylamide gels and then transferred to a 0.45 μm pore size nitrocellulose membrane (BA85; Schleicher and Schuell) using a Trans-Blot SD Semi-Dry Transfer Cell (Bio-Rad Laboratories) Nitrocellulose membranes were incubated in blocking solution consisting of 5% (w/v) skimmed milk powder in PBS/Tween-20 (0.1%, w/v) (PBS-T) for 1 h at room temperature, followed by incubation with the appropriate primary antibody diluted in blocking solution overnight at 4°C. Membranes were washed in PBS-T and then incubated with species-specific secondary antibodies in blocking solution for 1 h at room temperature in the dark, after which time the membranes were washed as above. Proteins were detected by scanning at 700 and 800 nm using the Odyssey detection system (LI-COR Biosciences).

##### Electron microscopy.

Aliquots of 2.5 μl of exosomes were placed on Formvar-coated grids and allowed to settle for 1–2 min without being allowed to dry. Exosomes were fixed with 2% glutaraldehyde for 5 min, washed 3 times with distilled deionized water, and then contrasted with 1 part 3% uranyl acetate in 9 parts 2% methyl cellulose for 10 min. Visualization of exosomes was by a FEI Tecnai T12 BioTWIN transmission electron microscope fitted with an AMT camera.

##### Immunocytochemistry/immunohistochemistry and antibodies.

Immunocytochemistry was performed as described previously (Goncalves et al., 2005). Paraffin wax-embedded spinal cord tissue was first dewaxed in xylene and 100% industrial methylated spirit (IMS, Sigma-Aldrich), heated in citric acid (10 mm, pH 6) until boiling, and then washed under a running tap for 5 min. The sections were washed 3× for 5 min each in PBS before incubation with primary antibody in PBS/0.02% Tween at 4°C overnight. Primary antibody was removed by washing 3× for 5 min each in PBS. The sections were incubated in the secondary antibody for 1 h at room temperature in PBS/0.02% Tween and then washed in PBS 3× for 5 min. Antibodies used were as follows: mouse monoclonal anti-βIII tubulin (Promega, 1:1000, catalog #G7121, RRID:AB_430874); chicken polyclonal anti-GFAP (Abcam, 1:300, catalog #ab4674, RRID:AB_304558); rabbit polyclonal anti-GFAP (DAKO, 1:2500,); mouse monoclonal anti-GFAP (Sigma-Aldrich, 1:100, catalog #Z0334, RRID:AB_10013382); rabbit polyclonal anti-RARβ (Santa Cruz Biotechnology, 1:100, catalog #sc-552, RRID:AB_2175379); rabbit polyclonal anti-NG2 (Millipore, 1:100, catalog #05-710, RRID:AB_309925); goat polyclonal anti-aldehyde dehydrogenase1A2 (Raldh2) (Santa Cruz Biotechnology, Inc, 1:100, catalog #sc-22591, RRID:AB_2224036); mouse monoclonal anti-CSPG (CS-56) (Sigma-Aldrich, 1:100, catalog #C8035, RRID:AB_476879), goat polyclonal anti-decorin (R&D Systems, 1:20, for immunochemistry and 5 μg/ml as a neutralizing antibody, catalog #AF143, RRID:AB_354790), rabbit polyclonal anti-decorin (Abcam, 1:50, catalog #ab175404), rat monoclonal anti-CD44 (eBioscience, 1:150, catalog #14-0441-86, RRID:AB_467248), mouse monoclonal anti-MAG (Abcam, 1:2000, catalog #ab89780, RRID:AB_2042411); goat polyclonal anti-Olig1 (Abcam, 1:1000, catalog #ab68105, RRID:AB_1142042); rabbit polyclonal anti-myelin PLP (Abcam, 1:1000, catalog #ab105784, RRID:AB_10973392); goat polyclonal anti-RARα (Abcam, 1:100, catalog #ab28767, RRID:AB_777684); rabbit polyclonal anti-OSP (Abcam, 1:2000, catalog #ab53041, RRID:AB_2276205); rabbit polyclonal anti-RA (Abnova, 1:300, catalog #PAB15482, RRID:AB_10759405); rabbit monoclonal anti-EGFR (Abcam, 1:500, catalog #ab52894, RRID:AB_869579); rabbit polyclonal anti-AIP1/Alix (1:1000 for Western blotting, catalog #ABC40, RRID:AB_10806218). Secondary antibodies for immunohistochemistry were Alexa Fluor 594 and Alexa Fluor 488 (1:1000, Invitrogen, Life Technologies) and Alexa Fluor 647 (1:1000, Invitrogen, Life Technologies). DAPI was used to stain nuclei (1 μg/ml, Sigma-Aldrich).

##### Confocal microscopy.

Multichannel fluorescence (DAPI–FITC–Texas Red filter set) images were captured using a Zeiss LSM 700 laser-scanning confocal microscope. For high-magnification images, a 63× oil-immersion Aprochromat objective (Zeiss) was used. Settings for gain, aperture, contrast, and brightness were optimized initially and held constant throughout each study so that all sections were digitized under the same conditions of illumination. Channels were imaged sequentially to eliminate bleed-through and multichannel image overlays were obtained using Adobe Photoshop version 7.0. Axiovision software was used to collect information on pixel immunoreactivity used for quantitative purposes.

##### Experimental design and statistical analysis.

Data and statistical analysis comply with the recommendations on experimental design and analysis in pharmacology (Curtis and Abernethy, 2015). Data analysis was performed in a blinded fashion. Group sizes were estimated based on previous experiments (Goncalves et al., 2015, 2018); eight rats per treatment condition were used for each experiment. All statistical analysis was performed using SigmaStat software (SPSS). Data are expressed as mean ± SEM. Histological data were analyzed using one-way or two-way repeated-measures ANOVA or Student's *t* test. *Post hoc* comparisons were performed where appropriate and all statistical tests are stated in the text. *Post hoc* tests were run only if *F* achieved *p* < 0.05 and when no significant variance in homogeneity was observed. Significance was accepted for *p* < 0.05. Exact *p*-values are shown except for ***p ≤ 0.001.

## Results

### RARβ signaling downregulates CSPGs by endogenous upregulation of decorin

We have used a model of DRA for our experimental *in vivo* studies. A DRA causes the disruption of the DR and contributing rootlets of the DREZ and injury to the dorsal column and horn along the SC segment connected to the avulsed root. This results in neurodegeneration, glial scar formation, and demyelination across the PNS-CNS interface, which a well delineated area, so this model offers a good pathological template to assess remyelination and the mechanisms involved.

**Figure 1.**
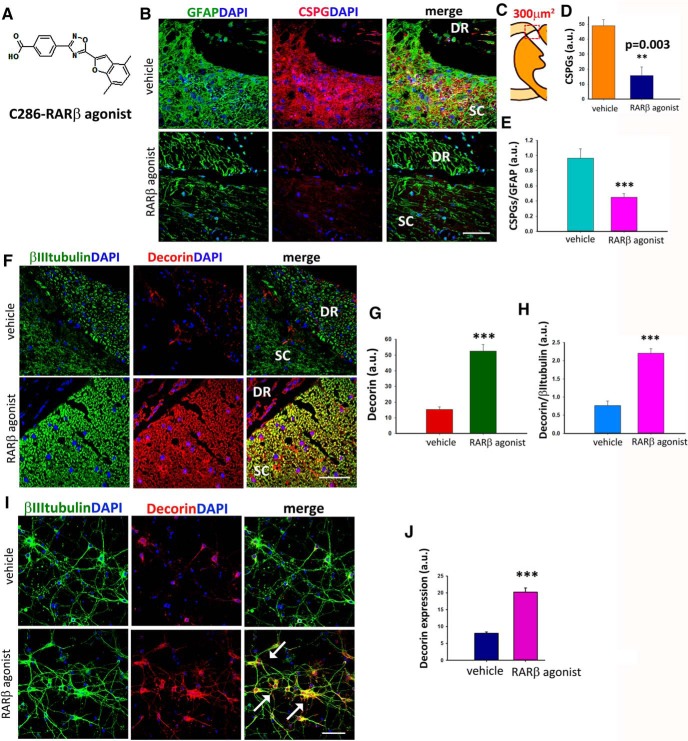
C286 reduces CSPGs expression and upregulates decorin expression after sensory root avulsion. ***A***, Chemical structure of the novel RARβ agonist C286. ***B***, Representative images of the expression of CSPG (CS-56) and GFAP at the PNS-CNS in injured vehicle- and C286-treated rats. ***C***, Representation of the area of the lesioned DREZ where the molecules of interest were quantified (300 μm^2^). ***D***, ***E***, Quantification of immunofluorescence intensity of total CS-56 (***D***) and CS-56 in astrocytes (***E***) shown in arbitrary units (a.u.) shows that CSPGs levels are significantly lower in C286-treated rats compared with vehicle-treated rats (*n* = 5 per treatment group, results are mean of fluorescence pixels ± SEM taken from 10 sections per animal, ***p ≤ 0.001, Student's *t* test). ***F***–***H***, Expression of decorin and βIII tubulin (***F***) and quantification of immunofluorescence intensity of total decorin (***G***) and decorin in neurons (***H***) shown in arbitrary units (a.u.) show that decorin is significantly higher in C286-treated rats compared with vehicle treated. Results are mean of fluorescence pixels ± SEM taken from 10 sections per animal and from five rats per treatment group, ***p ≤ 0.001, Student's *t* test. C286 induces upregulation of decorin in cultured neurons. ***I***, Expression of decorin in neurons cultured for 72 h with vehicle or C286 (10^−7^ m) is significantly higher in the latter (colocalization indicated by white arrows). Scale bar, 20 μm. ***J***, Quantification of decorin expression taken from 10 fields per culture condition from three independent experiments. Data represent mean of fluorescence intensity for decorin in arbitrary units (a.u.) ± SEM, ***p ≤ 0.001, Student's *t* test.

The accumulation of glial-scar-derived CSPGs at the injury site after SCI is a major obstacle to remyelination. CSPGs start being secreted in excess by reactive astrocytes within 24 h of the lesion and can persist for months afterward (Pindzola et al., 1993; Jones et al., 2003). We had previously observed remyelination of regenerated axons across the DREZ in this animal model after stimulation of RARβ signaling for 4 weeks. A possible mechanism whereby this pathway could be promoting remyelination is by CSPG clearance. To assess the effect, if any, of RARβ signaling on CSPGs, avulsed rats were treated orally with vehicle or a novel RARβ agonist C286 (Fig. 1*A*). Treatment was 3 mg/kg administered 3 times a week from day 2 after injury for 4 weeks because we found that this was the optimal dosing regimen to restore locomotor and sensory functions (Goncalves et al., 2019) that are stringently correlated with remyelination (McDonald and Belegu, 2006). We first analyzed the total CSPG levels determined by immunohistochemistry with CS56 antibody (Avnur and Geiger, 1984; Silver and Miller, 2004) from a 300 μm^2^ area of the DREZ (Fig. 1*B*,*C*). Vehicle-treated rats showed intense CS56 immunostaining [fluorescence intensity: 49 ± 3.93 arbitrary units (a.u.)] in the GFAP-enriched lesioned area, but the RARβ-treated rats had significantly lower levels of CS56 (fluorescence intensity: 15.75 ± 5.76 a.u.) (Fig. 1*D*). To assess the excess of CSPGs in astrocytes in the vehicle-treated rats compared with the rats treated with RARβ agonist, we calculated the ratios of fluorescent intensity of CSPGs/GFAP (Fig. 1*E*) and found that these were significantly higher in the vehicle-treated group (fluorescence intensity: 0.96 ± 0.35 a.u.) than in RARβ-treated rats (fluorescence intensity: 0.44 ± 0.13 a.u.). The modulation of CSPGs by an RARβ agonist had never been described before, so we next searched for molecular mechanisms that might explain this effect. Because decorin is modulated by RA (Pearson and Sasse, 1992) and is a potent endogenous CSPG scavenger (Esmaeili et al., 2014), we hypothesized that neuronal RARβ, which is upregulated by the RARβ agonist treatment in this animal model (Goncalves et al., 2018), could be inducing decorin. We assessed decorin expression in neurons immunohistologically (Fig. 1*F*). Quantification of total decorin in the same area used for CS56 analysis (Fig. 1*B*) was significantly higher in RARβ-treated rats (fluorescence intensity: vehicle, 15.25 ± 1.79 a.u.; RARβ agonist, 52.5 ± 4.17 a.u.). When the levels of βIII-tubulin, which were higher in RARβ-agonist-treated rats due to increased regeneration (Goncalves et al., 2015) were taken into account, the ratio decorin/βIII-tubulin was still significantly higher upon agonist treatment (fluorescence intensity: vehicle, 0.76 ± 0.31 a.u.; RARβ agonist, 2.2 ± 0.31 a.u.) (Fig. 1*G*,*H*), strongly suggesting that neuronal decorin is a major source of the RARβ-induced decorin upregulation.

To underpin a direct effect of neuronal RARβ signaling and decorin, we assayed for decorin expression in RARβ-agonist-treated neuronal cultures. Treatment with the agonist for 3 d significantly increased the expression of decorin compared with vehicle (fluorescence intensity: vehicle, 8 ± 0.4 a.u.; RARβ agonist, 20.25 ± 1.18 a.u.) (Fig. 1*I*,*J*).

**Figure 2.**
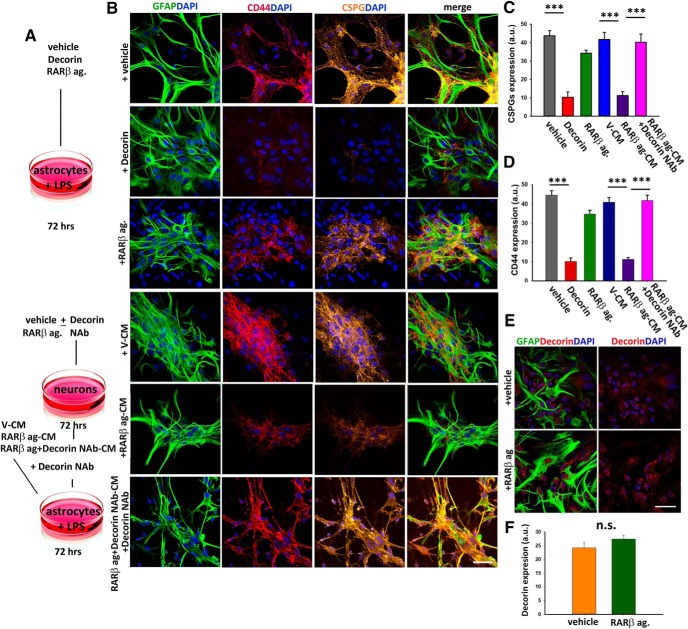
C286 suppresses CSPGs in reactive astrocytes via neuronal release of decorin. ***A***, Illustration of the cell culture treatments: LPS-activated astrocytes were treated with vehicle, decorin (20 μg/ml), or C286 or with conditioned medium from neurons previously treated with vehicle (V-CM) or C286 (C286-CM) in the presence or absence of decorin Nab, which was added to C286-treated neurons and astrocyte cultures that were treated with C286 + decorin NAb-CM. ***B***, Expression of CD44 and CSPGs in the astrocyte cultures. Scale bar, 20 μm. ***C***, ***D***, Decorin and C286-CM treatment significantly suppresses CSPGs and CD44, an effect that is abolished by the addition of decorin NAb to the cultures. Data represent mean of fluorescence intensity for CSPGs in arbitrary units (a.u.) ± SEM taken from 10 fields per culture condition from three independent experiments. ***p ≤ 0.001, one-way ANOVA followed by Fisher's *post hoc* test. ***E***, ***F***, LPS-activated astrocytes treated with vehicle or C286 and immunoassayed for GFAP (***E***) and decorin (***F***) showed no significant difference in decorin expression between the two treatments. Data represent mean of fluorescence intensity for decorin in arbitrary units (a.u.) ± SEM taken from 10 fields per culture condition from three independent experiments. n.s., Not significant, Student's *t* test.

Human recombinant decorin has been shown to inhibit inflammation, glial scar formation, and CSPG expression after SCI (Davies et al., 2004). To determine whether the neuronal RARβ-agonist-induced decorin release was sufficient to elicit anti-inflammatory effects and downregulation of CSPGs in reactive astrocytes, we cultured rat astrocytes and, after 24 h activation with LPS (Tarassishin et al., 2014), treated the cultures for 72 h with either decorin or RARβ agonist or conditioned media from neurons that had been treated with the agonist (RARβ ag-CM) or vehicle (V-CM) in the presence and absence of a decorin NAb (Li et al., 2008) (Fig. 2*A*). The cultures were then immunoassayed for total CSPGs (CS56) and for CD44, a transmembrane glycoprotein associated with reactive astrocytes (Girgrah et al., 1991). We found that there was a significant reduction of both CS56 and CD44 in the decorin (fluorescence intensity: CS56: 10.25 ± 3.03 a.u.; CD44:10.12 ± 1.92 a.u) and RARβ ag-CM (fluorescence intensity: CS56: 11.25 ± 2.13 a.u.; CD44:11.25 ± 0.92 a.u) treated cultures compared with vehicle (fluorescence intensity: CS56: 43.75 ± 2.78 a.u.; CD44: 44.57 ± 2.22 a.u), RARβ agonist (fluorescence intensity: CS56: 34.25 ± 1.65 a.u.; CD44: 34.75 ± 1.98 a.u), or V-CM (fluorescence intensity: CS56: 41.75 ± 3.9 a.u.; CD44: 40.87 ± 2.37 a.u) (Fig. 2*B–D*). To be certain that the reduction of CS56 and CD44 were a decorin-mediated effect, we repeated the experiment adding a decorin NAb to the cultures to block both exogenous and endogenous decorin because neurons and astrocytes can secrete this proteoglycan (Esmaeili et al., 2014). Remarkably, when the reactive astrocytes were treated with RARβ ag-CM in the presence of the decorin NAb, no significant decrease was seen on CS56 (fluorescence intensity: 40.25 ± 4.47 a.u) nor on CD44 (fluorescence intensity: 41.75 ± 2.37 a.u) (Fig. 2*B–D*). To determine whether the RARβ agonist had a direct action on the astrocytes to induce decorin secretion, we treated LPS-activated astrocytes with either vehicle or C286 for 72 h and then assessed the levels of decorin in the cultures (Fig. 2*E*,*F*). There was no significant difference between the two treatment groups (fluorescence intensity: vehicle, 24.2 ± 5.92 a.u.; RARβ agonist, 27.4 ± 4.32 a.u.). Together, this suggests that neuronal activation of RARβ induces decorin secretion from neurons that modulates the astrocytes inflammatory state and CSPG production.

### RARβ signaling activates RARα in regenerative myelination after DRA

**Figure 3.**
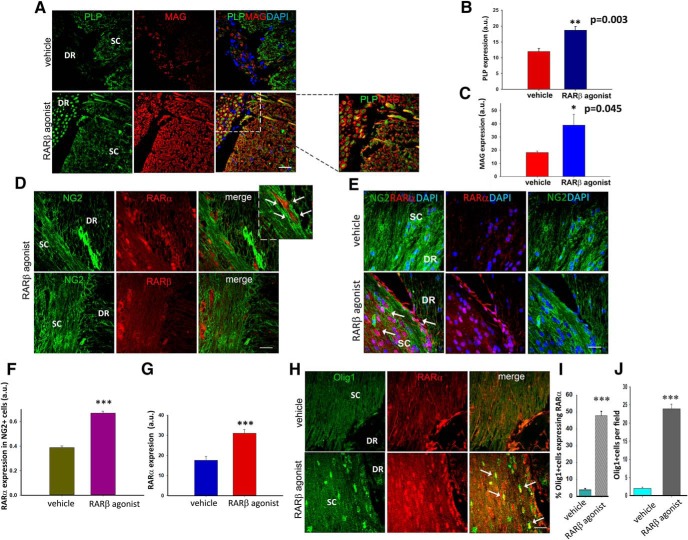
RARα-mediated myelination in RARβ-agonist-treated avulsed rats. ***A***, Expression of both PLP and MAG are upregulated in the RARβ-treated rats and both types of myelin show an unbroken pattern (inset). ***B***, ***C***, Quantification of PLP and MAG shows a significant increase in response to RARβ treatment. Data show mean ± SEM, *n* = 8 per treatment group, **p ≤ 0.01, Student's *t* test. ***D***, RARα, but not RARβ, is upregulated in NG2^+^ cells in the dorsal horn of RARβ-agonist-treated rats (see arrows). ***E***, Expression of RARα and NG2 in vehicle- and RARβ-agonist-treated rats shows an upregulation of RARα in the nucleus (seen in dark pink and indicated with white arrows) in C286-treated rats. ***F***, ***G***, Quantification of RARα in NG2 cells is significantly higher in this group (***F***) as is the total RARα expression (***G***) compared with vehicle-treated rats. Data are shown as mean ± SEM, *n* = 8 per treatment, ***p ≤ 0.001, Student's *t* test. ***H***–***J***, There is a high coexpression of Olig1 and RARα in RARβ-agonist-treated rats (***H***), with the percentage of Olig1^+^ cells expressing RARα (***I***) being significantly higher in this group compared with vehicle-treated rats, as is the total number of Olig1^+^ cells per field (***J***). Data are shown as mean ± SEM, *n* = 8 ***p ≤ 0.001, Student's *t* test. Scale bars, 100 μm.

To directly assess the effect RARβ signaling on remyelination *in vivo*, we compared the levels of myelination in the avulsed rats that had received vehicle or RARβ agonist treatment. We immunoassayed the injured SCs and dorsal roots for MAG, which is present in both the PNS and CNS (Quarles, 2002), and for PLP, which is the major myelin protein in the CNS (Kamholz et al., 1992). We had previously observed myelin sheaths around regenerated axons across the DREZ in response to RARβ activation using the same animal model (Goncalves et al., 2015). Unsurprisingly, we found that both PLP and MAG were significantly higher in RARβ-agonist-treated compared with vehicle-treated rats (fluorescence intensity: PLP: vehicle, 12 ± 0.91 a.u.; RARβ agonist, 18.75 ± 1.10 a.u.; MAG: vehicle, 18.22 ± 1.12 a.u.; RARβ agonist, 39 ± 8.13 a.u.) (Fig. 3*A–C*).

Because remyelination is preceded by the invasion and differentiation of oligodendrocyte progenitors at the lesion site, we investigated whether an increase in oligodendrocyte genesis could also contribute to the RARβ-agonist-induced remyelination observed. Previously, we had shown that RARα activation induces neural progenitor cell differentiation into oligodendrocytes (Goncalves et al., 2005) and RARα has been shown to be expressed in OPCs (Laeng et al., 1994), so we first looked at the expression of RARα and RARβ in the NG2^+^ cells in the injury area of myelinating rats. In agreement with previous *in vitro* observations (Laeng et al., 1994; Goncalves et al., 2005), we found that RARα was highly associated with NG2-immunoreactive cells, whereas little or no RARβ was detected in the NG2^+^ cells (Fig. 3*D*). We then compared the expression of RARα in NG2^+^ cells between vehicle-treated and RARβ-agonist-treated groups (Fig. 3*E*) and found that RARα immunostaining was significantly higher in NG2-expressing cells in the RARβ-agonist-treated group (fluorescence intensity: 0.66 ± 0.04 a.u.) compared with vehicle (fluorescence intensity: 0.38 ± 0.03 a.u.) (Fig. 3*F*). This was also true for total RARα expression (fluorescence intensity: vehicle, 17.63 ± 5.53 a.u.; C286, 30.99 ± 5.40 a.u.) (Fig. 3*G*). This suggests that RARβ drives RARα in this injury model and supports a function for RARα in promoting differentiation of the precursors into myelinating oligodendrocytes. Next, to determine whether the presence of RARα was associated with the differentiation process *in vivo*, we double stained tissue from myelinating and non-myelinating rats with RARα and Olig1, which is a transcription factor expressed from the early stages of oligodendrocyte lineage development (Woodruff et al., 2001) and essential to their differentiation into remyelinating oligodendrocytes (Arnett et al., 2004). Both RARα and Olig1 expression were significantly higher in the SCs of myelinating rats compared with non-myelinating and ∼50% of Olig1-expressing cells also expressed RARα (vehicle, 3.88 ± 0.73%; RARβ agonist 47.8 ± 2.65%), with the total number of Olig1^+^ cells at the DREZ in the-agonist-treated rats also being significantly higher (vehicle, 1.94 ± 1.10 and RARβ agonist, 23.88 ± 3.97 mean number of cells per field) (Fig. 3*H–J*), suggesting that this signaling pathway may be required throughout oligodendrocyte differentiation. Together, these data suggest that treatment with an RARβ agonist leads to an increase in myelinating oligodendrocytes driven by the removal of CSPGs and by the repopulation of oligodendrocytes at the lesioned site. However, the latter is not driven directly by RARβ, but rather by RARα, in the NG2^+^ cells.

### Intracellular calcium regulates RA associated exosome secretion in NG2^+^ cells

**Figure 4.**
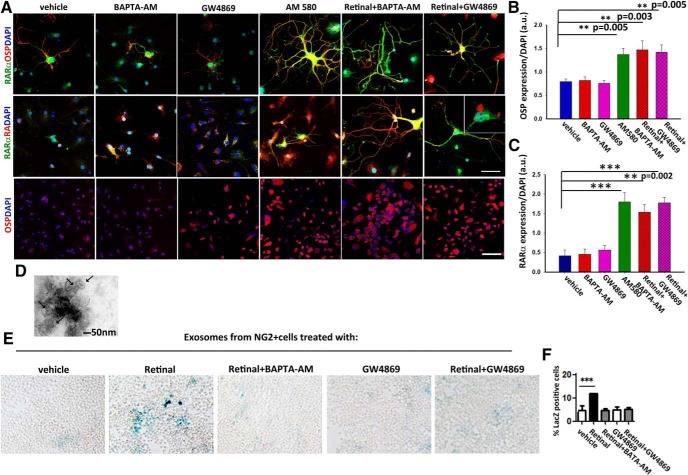
Intracellular calcium levels modulate the secretion of exosome-associated RA in NG2^+^ cells and determine their differentiation into oligodendrocytes via RARα signaling. ***A***, Expression of RARα, OSP, and RA in NG2 cultures treated for 3 d with: vehicle, BAPTA-AM, GW4869, AM 580, retinal + BAPTA-AM, or retinal + GW4869. Scale bar, 50 μm and for the bottom 300 μm. ***B***, ***C***, Quantification of OSP (***B***) and RARα (***C***) expression showed that both were significantly higher in the presence of AM580 or when increased intracellular concentrations of RA occurred (retinal + BAPTA-AM and retinal + GW4869). Data are shown as mean ± SEM taken from five random fields from three independent experiments, ***p ≤ 0.001, One-way ANOVA followed by Tukey's test. ***D***, Electron microscopy images of exosomes (arrows) isolated from vehicle-treated NG2^+^ cell cultures. Scale bar, 50 nm. ***E***, β-galactoside staining of RARE LacZ-transfected F9 cells showing RA in exosomes isolated from NG2^+^ cell cultures treated with: vehicle, retinal, retinal + BAPTA-AM, GW4869, or retinal + GW4869. ***F***, Quantification of RA plotted as percentage of LacZ^+^ cells shows that preventing exosome release from the NG2^+^ cells results in a significant decrease in secreted RA. Data are shown as mean ± SEM taken from five random fields from three independent experiments, ***p ≤ 0.001, one-way ANOVA, followed by Tukey's test.

The above results pose the question: how are these two signaling pathways connected? We had previously shown that treatment of avulsed rats with the RARβ agonist leads to an increase of Raldh2 activity in the NG2^+^ cells that populate the injury site and an increase in the release of RA associated with exosomes that serve as guidance cues for the regenerating axons (Goncalves et al., 2018). Because we did not administer an RARα agonist in addition to the RARβ agonist, we reasoned that RA as the endogenous ligand had to be driving RARα signaling in the NG2^+^ cells. A shift in RA traffic by curtailing exosome release could be the cause of RARα activation. Calcium is known to govern exosome release, with increased intracellular concentrations inducing its secretion (Savina et al., 2003). To determine whether a change in intracellular calcium and exosome release was involved in a RARα-mediated NG2^+^ cell differentiation, we treated NG2^+^ cells with vehicle, a calcium chelator (BAPTA-AM), or GW4869, which is the most widely used pharmacological agent for blocking exosome biogenesis/release (Essandoh et al., 2015), a specific RARα agonist (AM 580), or BAPTA-AM plus retinal to provide the substrate for RA synthesis, or retinal plus GW4869. After 3 d in culture, we looked at the oligodendrocyte marker, OSP (Sallis et al., 2006) and RARα expression. Only AM 580 and BAPTA-AM plus retinal or retinal plus the exosome inhibitor resulted in the upregulation of OSP and RARα (fluorescence intensity: OSP- vehicle, 0.78 ± 0.06 a.u.; BAPTA-AM, 0.81 ± 0.07 a.u.; GW4869, 0.75 ± 0.09 a.u.; AM 580, 1.37 ± 0.13 a.u.; retinal+ BAPTA-AM, 1.46 ± 0.19; retinal+ GW4869, 0.59 ± 0.05 a.u.; RARα - vehicle, 0.41 ± 0.29 a.u.; BAPTA-AM, 0.45 ± 0.26 a.u.; GW4869, 0.56 ± 0.23 a.u.; AM 580, 1.80 ± 0.47 a.u.; retinal+ BAPTA-AM, 1.53 ± 0.38; retinal+ GW4869, 0.77 ± 0.27 a.u.) (Fig. 4*A–C*). In the treatments that lead to an increase in OSP, we also observed an intracellular increase in RA (Fig. 4*A*, middle), possibly due to cytoplasmic accumulation because it has not been secreted. To confirm that the NG2^+^ cells retain intracellular RA that would otherwise be secreted in association with exosomes as a response to intracellular calcium changes, we isolated exosomes as described previously (Goncalves et al., 2018) from NG2^+^ cells cultures treated as above. To obtain confirmation of successful isolation, exosomes were imaged using electron microscopy. A representative photograph of an exosome pool isolated from a vehicle-treated culture is shown in (Fig. 4*D*), where various microvesicles with a size in the range of 50–100 nm could be seen, which is consistent with the exosome size (Kastelowitz and Yin, 2014). After 3 d in culture, exosomes were isolated and placed on F9 RARE-lacZ RA reporter cells as described previously (Goncalves et al., 2018). We found that the retinal induced release of RA and this was blocked by the presence of BAPTA-AM and GW4869 to a similar extent (percentage of LacZ^+^ cells: vehicle, 4.8 ± 0.94; retinal, 11.98 ± 0.02; retinal+ BAPTA-AM, 4.65 ± 0.16; GW4869, 5.12 ± 0.57; retinal+ GW4869, 5.22 ± 0.25) (Fig. 4*E*,*F*). Collectively, these data support a role for intracellular calcium concentrations in governing the release of exosomes containing RA from the NG2^+^ cells.

### Decorin regulates the release of exosome-associated RA in NG2^+^ cells via the EGFR–calcium pathway

**Figure 5.**
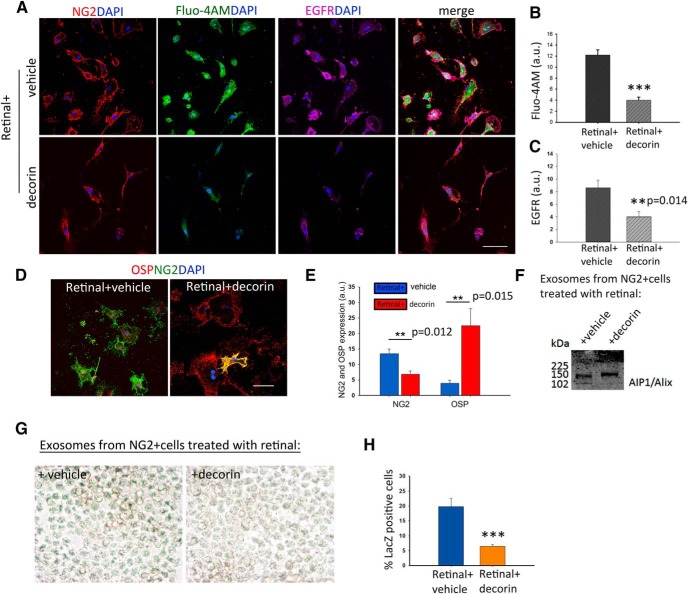
Decorin drives NG2^+^ cells differentiation into oligodendrocytes through control of RA-associated exosome release via the EGFR–calcium pathway. ***A***, NG2^+^ cells cultured on a CSPG substrate in the presence of retinal or retinal with decorin for 3 h show a downregulation of EGFR and intracellular Ca^2+^ (Fluo-4AM) in the decorin-treated cultures. ***B***, ***C***, Quantification of Fluo-4AM and EGFR immunofluorescence in the cultures. ***D***, Expression of OSP in the same cultures. ***E***, Quantification of NG2 and OSP^+^ cells show that decorin enhances significantly the differentiation of NG2^+^ cells to oligodendrocytes. Data represent mean of fluorescence intensities in arbitrary units (a.u.) ± SEM calculated from five fields per culture condition from three independent experiments, **p ≤ 0.005, ***p ≤ 0.001, Student's *t* test. ***F***, Western blot of exosomes isolated from retinal + vehicle- and retinal + decorin-treated cultures with AIP1/Alix antibody. ***G***, β-galactoside staining of RARE LacZ transfected F9 cells showing RA in exosomes isolated from NG2^+^ cell cultures treated with: vehicle + retinal and decorin + retinal. ***H***, Quantification of RA shown as percentage of LacZ^+^ cells suggests that decorin prevents the secretion of RA in exosomes. Data are shown as mean ± SEM obtained from five fields per culture treatment from three independent experiments, ***p ≤ 0.001, Student's *t* test. Scale bars, 50 μm.

Decorin has been shown to downregulate EGFR within 3 h (Zhu et al., 2005). EGFR activation can induce Ca^2+^ release from endoplasmic reticulum stores (Wang et al., 2005), thus increasing intracellular Ca^2+^ concentrations. To determine whether decorin could regulate exosome release from the RA synthesizing NG2^+^ cells by inducing changes in intracellular Ca^2+^, we cultured NG2^+^ cells on a CSPG matrix to mimic the extracellular milieu after a SCI using retinal as the Raldh2 substrate and treated the cultures with vehicle or decorin. After 2 h, we added a Ca^2+^ dye (Fluo-4AM) to the medium and allowed the cells to incubate for another hour before assessing the levels of intracellular Ca^2+^ and EGFR by immunocytochemistry. We found that decorin significantly reduced intracellular Ca^2+^ (fluorescence intensity: retinal + vehicle, 12.2 ± 0.91 a.u.; retinal + decorin, 4 ± 0.83 a.u.) and EGFR (fluorescence intensity: retinal + vehicle, 8.6 ± 1.2 a.u.; retinal + decorin, 4 ± 0.54 a.u.) (Fig. 5*A–C*). To evaluate the effect of decorin on oligodendrocyte differentiation, we used cultures treated the same way and looked at the expression of NG2 and OSP after 3 d in culture. Decorin significantly increased the differentiation of NG2^+^ cells (fluorescence intensity: retinal + vehicle, 13.48 ± 1.53 a.u.; retinal + decorin, 6.85 ± 1.06 a.u.) into OSP^+^ cells (fluorescence intensity: retinal + vehicle, 3.9 ± 10.97 a.u.; retinal + decorin, 22.52 ± 5.43 a.u.) (Fig. 5*D*,*E*). Finally, to directly assess the effect of decorin in the exosome-associated RA release, we collected the conditioned media from these cultures and isolated exosomes as described previously (Goncalves et al., 2018). Western blotting with the exosome marker AIP1/Alix (Willms et al., 2016) was used to confirm exosome isolation (Fig. 5*F*). These were then added to F9 RARE-lacZ RA reporter cells as described above for quantification of RA. As anticipated, we found that exosomes from decorin-treated NG2^+^ cells had significantly lower levels of RA than retinal plus vehicle ones, as shown by the percentage of LacZ^+^ cells extent (percentage of LacZ^+^ cells: retinal + vehicle, 419.81 ± 2.8; retinal + decorin, 6.43 ± 0.72) (Fig. 5*G*,*H*).

### RARα is required for the differentiation of NG2^+^ cells into oligodendrocytes

**Figure 6.**
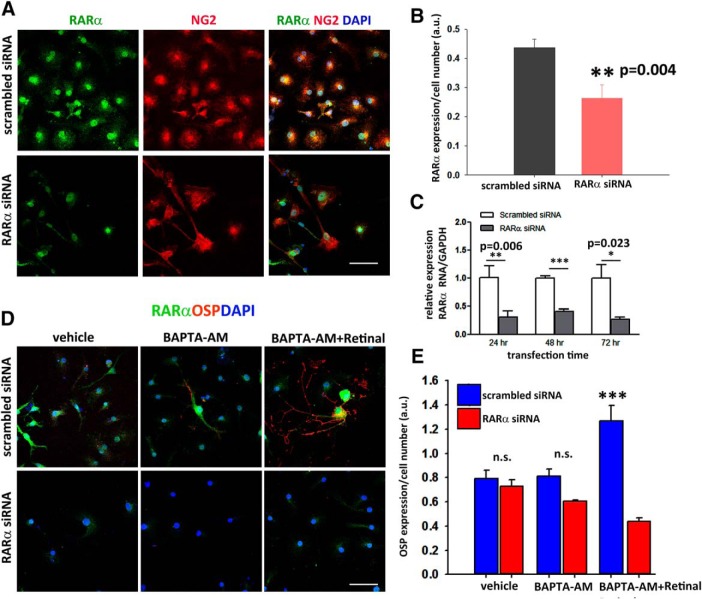
RARα is required for the differentiation of NG2^+^ cells into oligodendrocytes. ***A***, Immunostaining of NG2^+^ cell cultures transfected with scrambled or RARα siRNA showing that transfection with RARα siRNA resulted in the loss of RARα. ***B***, Quantification of RARα expression normalized for cell number (a.u.). Data are shown as mean ± SEM, **p ≤ 0.01, Student's *t* test. ***C***, Quantitative analysis of RNA levels normalized to GAPDH by qRT-PCR confirm the suppression of RARα over 72 h. Data are shown as mean ± SEM obtained from five fields per culture condition from three independent experiments, *p ≤ 0.05, **p ≤ 0.01, ***p ≤ 0.001, Student's *t* test. ***D***, Expression of RARα and OSP in scrambled and RARα siRNA-transfected NG2^+^ cells in the presence of vehicle, BAPTA-AM, or BAPTA + retinal. Only scrambled siRNA-transfected NG2^+^ cells treated with BAPTA-AM + retinal differentiate into oligodendrocytes. ***E***, Quantification of OSP^+^ normalized for number of cells in the cultures taken from five fields per culture condition from three independent experiments, Data are shown as mean ± SEM, ***p ≤ 0.001. n.s., Not significant, Student's *t* test. Scale bars, 50 μm.

Next, to confirm the requirement of RARα signaling to drive the differentiation process, we transfected NG2^+^ cells with an RARα siRNA or a scrambled siRNA and cultured them with vehicle, BAPTA-AM, or BAPTA-AM plus retinal for 4 d. First, we confirmed that RARα was knocked down by immunocytochemical RARα expression (fluorescence intensity: scrambled siRNA, 0.43 ± 0.02 a.u.; RARα siRNA, 0.26 ± 0.04 a.u.) (Fig. 6*A*,*B*), and by qPCR of RARα over 72 h (relative expression of RARα RNA/GAPDH: 24 h, scrambled siRNA, 1.0 ± 0.20 and RARα siRNA 0.3 ± 0.11; 48 h, scrambled siRNA, 0.99 + 0.04 and RARα siRNA 0.4 + 0.04, 72 h, scrambled siRNA, 0.76 ± 0.23 and RARα siRNA, 0.27 ± 0.03) (Fig. 6*C*). We next looked at the differentiation into oligodendrocytes in the same culture conditions. In RARα siRNA-transfected NG2^+^ cells, treatment with BAPTA-AM and retinal yielded a significantly lower number of OSP^+^ cells than in the scrambled or nontransfected cultures (fluorescence intensity: scrambled siRNA: vehicle, 0.79 ± 0.07 a.u.; BAPTA-AM, 0.81 ± 0.06 a.u.; BAPTA-AM+ retinal, 1.26 ± 0.12 a.u. RARα siRNA; vehicle 0.72 ± 0.05 a.u.; BAPTA-AM, 0.6 ± 0.01 a.u.; BAPTA-AM+ retinal, 0.43 ± 0.02 a.u.) (Fig. 6*D*,*E*). This suggests that RARα plays a crucial role in the differentiation of NG2^+^ cells into oligodendrocytes.

### Raldh2 activity is required for remyelination *in vivo*

**Figure 7.**
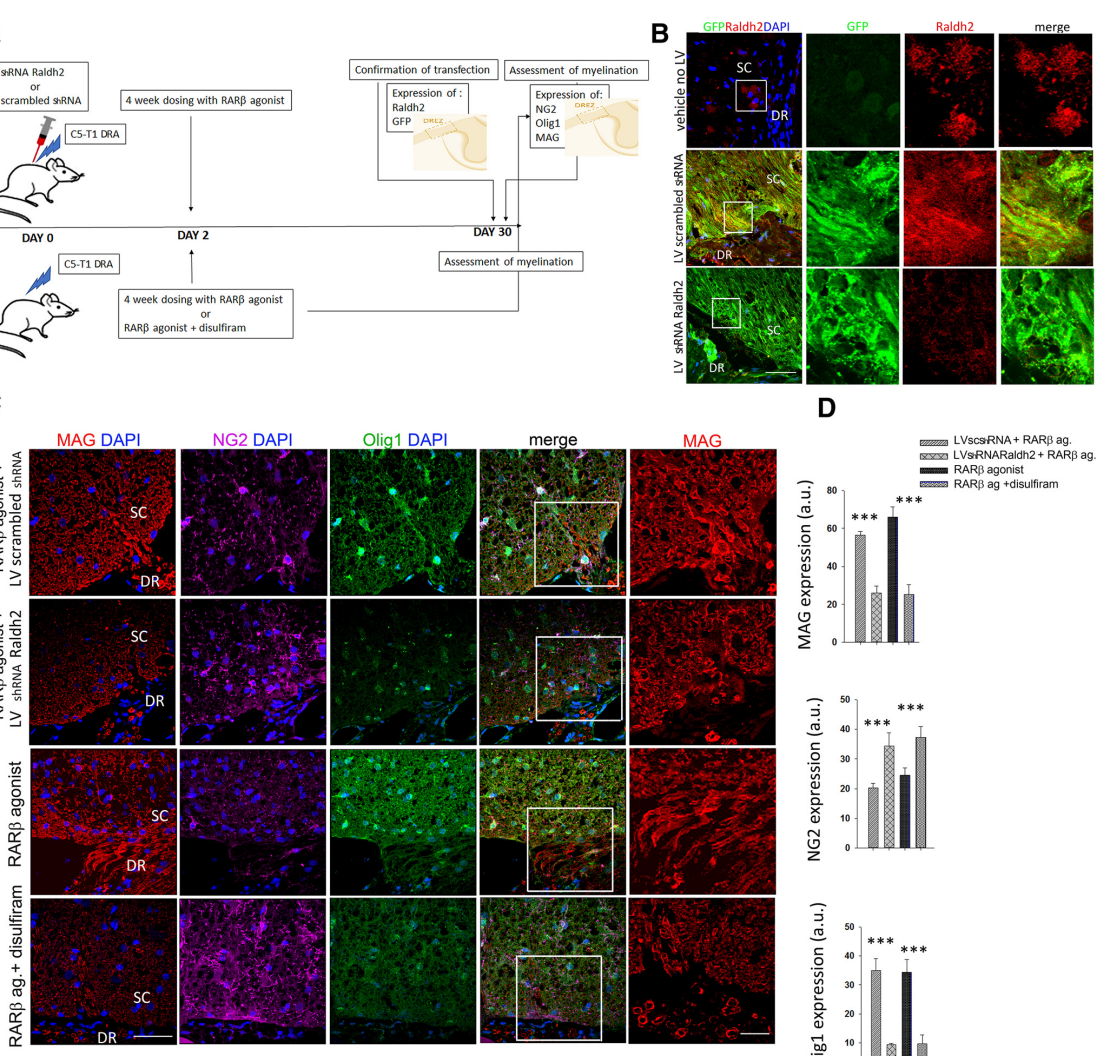
Raldh2 activity is required for differentiation of NG2^+^ cells into myelinating oligodendrocytes after DRA. ***A***, Experimental design. Four groups of rats were subject to C5–T1 DRA. In two groups, an LV was injected at the lesion site (DREZ) at the time of surgery; one was an LV shRNA Raldh2 and the other a scrambled shRNA. All rats were treated for 4 weeks from day two after the surgery. All LV-injected rats received RARβ agonist (3× a week), another group received RARβ agonist only, and the fourth group received RARβ agonist and disulfiram. At the end of the treatment period, SCs with corresponding injured DRs were collected for protein expression analysis. LV transfection was confirmed by GFP and Raldh2 immunostaining from a subgroup of rats culled 2 weeks after injury. At the end of the treatment period, myelination was assessed by the quantification of NG2, Olig1, and MAG at the DREZ. ***B***, Images of the DREZ from a control group that had not been transfected with LV. LV-transfected rats show expression of Raldh2 and GFP in the scrambled LV and very little of Raldh2 in shRNA Radh2 LV. Scale bar, 50 μm. ***C***, Expression of MAG, NG2, and Olig1 at the DREZ in all four treatment groups. Scale bars, 50 and 20 μm for left inset images. ***D***, Quantification of immunofluorescence expressed in arbitrary units (a.u.) taken from an area of 300 μm^2^ comprising the DREZ and adjacent PNS and CNS areas shows that MAG and Olig1 are significantly upregulated in scrambled LV- and RARβ-agonist-treated rats compared with Raldh2 shRNA LV- and RARβ agonist + disulfiram-treated rats. NG2 was significantly higher in these groups. Results are mean of fluorescence pixels ± SEM taken from 10 sections per animal from five rats per treatment group, **p ≤ 0.005, ***p ≤ 0.001, Student's *t* test.

Our results thus far suggest that the differentiation of NG2^+^ cells into myelin-producing oligodendrocytes is reliant upon the synthesis of RA as the endogenous ligand of RARα. We took a pharmacological and functional approach to confirm this. We performed the DRA as described above and knocked down Raldh2 by injecting at the DREZ a GFP-tagged lentivirus with an shRNA to silence Raldh2 (LV shRNA Raldh2) or an LV-overexpressing a scrambled shRNA (LV scrambled) at the time of the injury and then treated both groups with the RARβ agonist as before for 4 weeks. A separate set of avulsed rats were given a Raldh2 inhibitor, disulfiram, together with the RARβ agonist or vehicle for 4 weeks after the injury (Fig. 7*A*). For the LV-injected rats, we first confirmed LV transduction by assessing GFP and Raldh2 expression in the lesioned area after 2 weeks of LV injections. As a control we have included a lesioned, vehicle-treated rat which had not been injected with the LV. In LV shRNA Raldh2 RARβ-agonist-treated rats there was hardly any colocalization of Raldh2^+^ staining with GFP, unlike the LV scrambled shRNA RARβ-agonist-treated rats where several areas of colocalization could be seen at the DREZ (Fig. 7*B*), suggesting a successful LV transduction. Next, to evaluate the effect of Raldh2 on myelination, MAG, NG2, and Olig1 expression at the DREZ area were assessed at the end of the treatment period (Fig. 7*C*). Both the LV shRNA Raldh2 + RARβ agonist and RARβ agonist + disulfiram-treated groups showed significantly less MAG and Olig1 than the LV-scrambled shRNA + RARβ agonist and RARβ-agonist-treated groups (fluorescence intensity for MAG: LV scrambled shRNA+ RARβ agonist, 56.43 ± 1.93 a.u.; LV shRNA Raldh2+ RARβ agonist 25.98 ± 3.75 a.u.; RARβ agonist, 65.90 ± 5.55 a.u.; RARβ agonist+ disulfiram 25.16 ± 5.14 a.u. fluorescence intensity for Olig1: LV scrambled shRNA+ RARβ agonist, 34.95 ± 4.12 a.u.; LV shRNA Raldh2+ RARβ agonist, 9.41 ± 0.44 a.u.; RARβ agonist, 34.41 ± 4.37 a.u.; RARβ agonist+ disulfiram 9.71 ± 3 a.u.) (Fig. 7*D*). Conversely, the groups in which Raldh2 had been suppressed showed a higher expression of NG2^+^ cells at the injury site (fluorescence intensity: LV scrambled shRNA+ RARβ agonist, 20.27 ± 1.55 a.u.; LV shRNA Raldh2+ RARβ agonist, 34.38 ± 4.44 a.u.; RARβ agonist, 24.56 ± 2.48 a.u.; RARβ agonist+ disulfiram 37.29 ± 3.63 a.u.) (Fig. 7*D*), suggesting that these cells remained at an early lineage stage of development and did not differentiate into oligodendrocytes.

**Figure 8.**
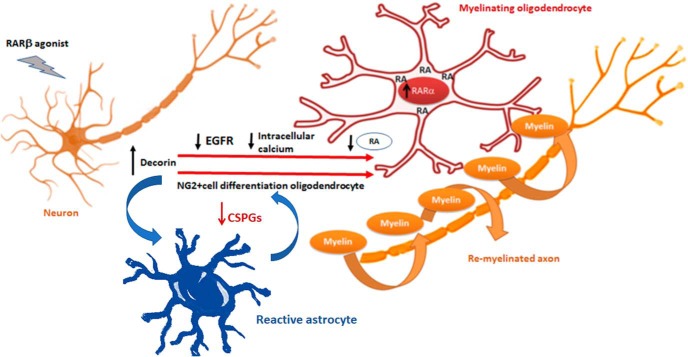
Decorin and RA signaling role in NG2^+^ cell differentiation into myelinating oligodendrocytes. Schematic representation of the proposed interplay between decorin- and RARα-mediated differentiation of NG2^+^ cells into myelinating oligodendrocytes. Activation of RARβ in neurons results in the upregulation of decorin, which is released and downregulates the EGFR in NG2^+^ cells. This leads to a decrease in intracellular calcium. The NG2^+^ cells that are synthesizing RA will cease/decrease RA secretion in exosomes due to the lowering of intracellular calcium and this will lead to an increase of intracellular RA to activate RARα signaling that drives differentiation into myelinating oligodendrocytes. Additionally, decorin decreases the CSPGs derived from reactive astrocytes and thus tackles a major obstacle to myelination.

Our results support a model (Fig. 8) whereby RARβ activation in neurons leads to the increased synthesis and secretion of decorin. The decorin has a twofold promyelinating effect: (1) it acts as a scavenger of the CSPGs produced by reactive astrocytes, thus eliminating a major obstacle to OPCs differentiation and migration, and (2) its action on NG2^+^ cells that are synthesizing RA is to suppress EGFR and reduce intracellular calcium concentrations. This restricts exosome release and generates an intracellular pool of RA, which activates RARα signaling and promotes differentiation into myelinating OLs.

## Discussion

We show here that RA signaling promotes remyelination after SCI through the synergic interaction of neuron–glia network pathways. In this context, we identify decorin as an important downstream target of RARβ in neurons. The well documented effect of decorin on CSPGs is at least one of the ways by which the RARβ agonist induces CSPG clearance. The inhibitory effect of CSPGs on OPC migration and differentiation has been associated with the downregulation of the β1-integrin pathway (Sun et al., 2017). Several approaches to neutralizing CSPGs after injury have included digestion of CSPGs with the enzyme chondroitinase ABC (Moon and Fawcett, 2001; Bradbury et al., 2002), RNA interference of chain polymerization enzyme (Laabs et al., 2007), and peptide blocking of protein tyrosine phosphatase σ (Lang et al., 2015). However, modulating CSPGs via the activation of an endogenous pathway offers clear therapeutic advantages.

Additionally, we found that the RARβ–decorin downstream cascades are not limited to astrocytes, but interestingly and crucially for remyelination, extend to NG2^+^ cells. Treatment with RARβ agonist leads to the upregulation of Raldh2 in the NG2^+^ cells that populate the injury site (Goncalves et al., 2018). During axonal regeneration, which precedes myelination, the RA is preferentially secreted in association with exosomes to act as an axonal pathfinder (Goncalves et al., 2018). We found that decorin significantly suppresses RA release via an EGFR-mediated decrease in intracellular calcium. In agreement with our data, decorin has been shown to attenuate the EGFR-mediated mobilization of intracellular calcium (Csordás et al., 2000). Analysis of EGFR in the neonatal SVZ progenitor cells showed that a loss of the receptor occurs as the cell lineage is established and very few NG2^+^ and Olig2^+^ cells expressed EGFR (Cesetti et al., 2009).

We found that the reduction of secretion of exosome-associated RA results in the activation of RARα in the NG2^+^ cells and in an increase in oligodendrocyte biogenesis. RARα has been shown to be constitutively present in OPCs and to be transiently increased by RA (Laeng et al., 1994), probably RARα2, because only this isoform has been reported to be induced by RA (Leroy et al., 1991), whereas RARβ is not constitutively expressed in OPCs (Laeng et al., 1994). However, prolonged treatment of human-derived OPCs with RA results in RARβ upregulation and in the upregulation of two known transcriptional inhibitors of oligodendrocyte differentiation, Hes5 and Id4 (Kim et al., 2017). In embryonic SC, where relatively high levels of retinoids are present, RA was found to inhibit oligodendrocyte differentiation during early embryonic development, permitting OPCs dispersal throughout the entire SC (Noll and Miller, 1994). Although we did not directly measure intracellular RA, it is likely that a fluctuation of RA levels, tightly modulated by neuron–glia signals, will provide the stop–go instructions to the NG2^+^ cell via RAR expression to keep the OPC/OL pools under control. It is thus thought that the developmental-stage-specific actions of RA are modulated through the spatiotemporal expression of the subtype and isoforms of RARs (Takeyama et al., 1996), as well as by RA gradients (Maden et al., 1996, 1998), and a similar mechanism may apply to the adult regenerating nervous system.

Extracellular vesicles are important in neuron–glia communications (for review, see Frühbeis et al., 2013) and specifically exosome signaling has been shown to play a role in the demyelinating disease multiple sclerosis (Carandini et al., 2015; Pusic et al., 2016). We propose that, like myelination in development, the dynamic cross talk between neurons and oligodendrocytes adapts to the physiological requirements and this is reflected in the cues exchanged (Simons and Trajkovic, 2006). The current study does not specifically address the timing for the switch between release and nonrelease mechanisms of RA associated with exosomes from the NG2^+^ cells and it is possible that they are both present during the 4-week period of RARβ agonist treatment, but not in individual NG2^+^ cells, according to the state of regeneration of the axons in their vicinity.

The importance of retinoid signaling in myelination has been previously shown (Huang et al., 2011; Kazakova et al., 2006; Latasa et al., 2010) and has been proposed to be mediated by RXRS (Huang et al., 2011). However, the identification of endogenous ligands for RXRs is contentious (Wolf, 2006; Gilardi and Desvergne, 2014). RXRs are partners to a number of other nuclear receptors (Lefebvre et al., 2010), thus integrating the corresponding signaling pathways. We have identified that RARα promotes remyelination. Our findings, together with previous work (Huang et al., 2011), indicate that the RARα/RXR pathway is a promising avenue for remyelination strategies.

Collectively, our study sheds important insights into the neuron–glia networks during remyelination. Our data suggest that targeting the RA signaling may overcome the myelination-inhibitory environment that persists after SCI as well as promoting myelination per se. Various drugs have been identified that promote myelination (Mei et al., 2014; Najm et al., 2015; Keough et al., 2016), but these drugs may not overcome inhibition by extrinsic factors in the demyelinated lesions. Similarly, OPC differentiation drugs failed to rescue inhibition of their differentiation by extracellular CSPGs (Keough et al., 2016), suggesting a need for new approaches to target extrinsic inhibition of remyelination alongside oligodendrocyte genesis. Additionally, and most relevant from a therapeutic view point, we show here that an orally available RARβ agonist can induce endogenous synthesis of RA and subsequent RARα activation in NG2^+^ cells in the nervous system, leading to regenerative myelination. This offers an advantageous strategy to promote myelination in neurological diseases with CSPG deposition. The novel RARβ agonist used in the experiments described here is orally available and has excellent potency (RARβ EC_50_ = 1.94 nm) and selectivity for RARβ (RARα/RARβ ratio = 13.4; RARγ/RARβ ratio = 5.6). C286 is currently being tested in a phase 1 trial (ISRCTN12424734), which, if successfully completed, confers a tangible translatable aspect to the work presented herein.
